# The Effect of Additional Private Health Insurance on Mortality in the Context of Universal Public Health Insurance

**DOI:** 10.3390/ijerph18168363

**Published:** 2021-08-07

**Authors:** Eun-Mi Baek, Jae-Il Oh, Eun-Jung Kwon

**Affiliations:** 1 Department of Preventive Medicine, College of Medicine, Catholic University of Korea, Seoul 06591, Korea; hanel2004@naver.com (E.-M.B.); kwonruby@naver.com (E.-J.K.); 2 Bagae Hospital Health Promotion Center, Pyeongtaek 17909, Korea

**Keywords:** national health insurance (NHI), private health insurance (PHI), universal health coverage (UHC)

## Abstract

(1) Background: Korea operates its national health insurance (NHI) system as a form of public health insurance, and is commonly regarded as having achieved universal health coverage (UHC). However, many Korean households register for additional private health insurance (PHI) programs. Typically, registration rates for PHI are higher for individuals with a higher socioeconomic status (SES). A difference in mortality between those with and without additional PHI would indicate that there are health inequalities within the Korean NHI system under UHC. Therefore, this study aimed to confirm whether additional PHI affects mortality under the Korean NHI system. (2) Methods: We conducted a longitudinal study using the Korean Longitudinal Study of Aging data from the first to the sixth wave. The analysis included 8743 participants, who were divided into two groups: those who only had NHI and those who had both NHI and PHI. Differences in mortality between the two groups were compared using the Cox proportional hazard regression. (3) Results: The group with both NHI and PHI had lower mortality than the group with only NHI (hazard ratio = 0.53, 95% confidence interval: 0.41, 0.9). (4) Conclusions: The results of this study reveal that there are health disparities according to SES and PHI within the Korean NHI system under UHC. Therefore, relevant government institutions and experts should further improve the NHI system to reduce health disparities.

## 1. Introduction

Different countries design and operate public health insurance systems in accordance with their level of economic prosperity and their social environment, with rates of health insurance coverage varying from country to country. Korea currently operates the National Health Insurance (NHI) program, a public health insurance system similar to those operated in Germany, France, the United Kingdom, and Japan [1,2]. The World Health Organization (WHO, Geneva, Switzerland) defines universal health coverage (UHC) as universal access to key promotive, preventive, curative, and rehabilitative health interventions at an affordable cost [3]. Korea has expanded its NHI coverage on the basis of rapid economic growth and is currently regarded as having achieved UHC. As outlined in the WHO definition of UHC, public health insurance subscribers under UHC are required to bear a portion of the costs, and some private medical costs can fall outside the scope of public health insurance. Member states of the Organization for Economic Co-operation and Development typically cover approximately 70% of their citizens′ total medical costs through public health insurance [4]. The Korean situation is similar, with 20% of all medical costs being borne by the patient in the case of hospitalization, although the costs of medical services that are not covered by the NHI, such as new medical technologies and new drugs, are borne entirely by the patient. NHI in Korea does not have a system that reimburses patients for expenses after a health technical assessment (HTA).

Some individuals purchase private health insurance (PHI) to prepare for the economic costs incurred when accessing such medical services. In Korea, despite the fact that all citizens are covered by NHI, more than 75% of households have additional PHI, which amounts to a collective spend of more than KRW 24 trillion per year [5]. To purchase PHI, participants are required to have the financial means to bear additional insurance costs. Older individuals and people with past medical histories often face higher insurance costs or other difficulties when acquiring PHI [6]. If mortality differs depending on PHI status in this context, this would suggest that there are health inequalities that result in varying degrees of coverage according to socioeconomic status (SES) [7,8,9,10,11]. Very few studies have examined whether PHI registration influences mortality under a public health insurance system that provides UHC. Therefore, this study aimed to confirm whether having additional PHI affects mortality for people enrolled on the Korean NHI system under UHC. We examined NHI subscribers by analyzing data from the Korean Longitudinal Study of Aging (KLoSA).

## 2. Materials and Methods

This was a longitudinal study using data from the first to the sixth wave of the KLoSA, a panel survey conducted by the Korea Labor Institute and the Korea Employment Institute Information Service. The KLoSA was conducted for the first time in 2006 for the purpose of collecting basic data for designing and implementing effective socioeconomic policies related to aging; it included 10,254 individuals from 6171 households. The KLoSA has been conducted every 2 years since then and was conducted for the sixth time in 2016. The survey population comprises Korean citizens residing in Korea aged 45 or older. Sampling was performed by extracting 1000 sample study areas across Korea and using simple random sampling to select households in those 1000 study areas. Households were eligible for the study if at least one individual aged 45 or older lived there. Surveys were conducted by interviewers who visited the selected households in a set order and subsequently interviewed all members of the household aged 45 or older. A total of 10,254 individuals were interviewed in the first wave of the survey, 8875 in the second wave, 8229 in the third wave, 7813 in the fourth wave, 7467 in the fifth wave, and 7015 in the sixth wave. The sample retention rates were 86.6% for the second wave, 81.7% for the third wave, 80.1% for the fourth wave, 79.2% for the fifth wave, and 78.0% for the sixth wave. Interviews were conducted using a computer-assisted personal interviewing method. This study was conducted after receiving approval from the Institutional Review Board of Catholic University (MC19ZESI0077).

### 2.1. Study Population

**Figure 1:**
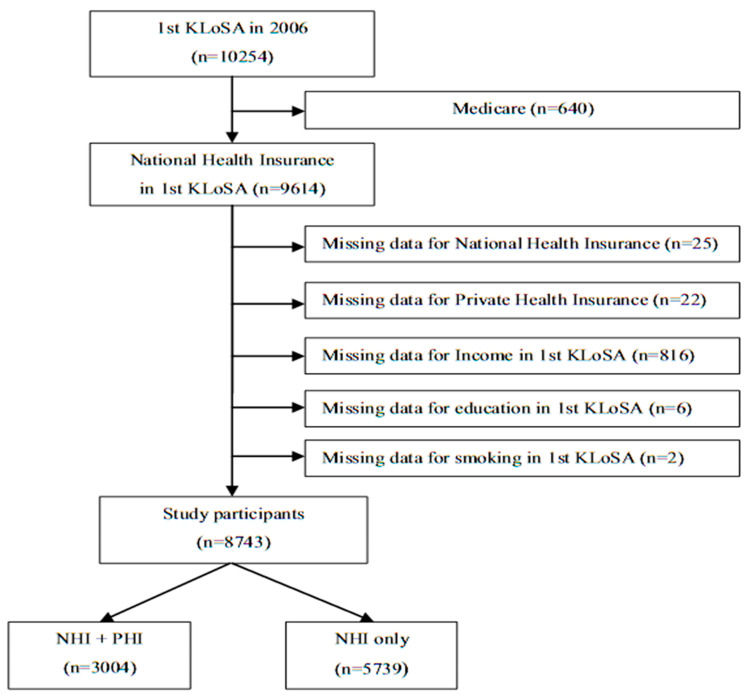
Selection of study participants. Abbreviations: NHI = National Health Insurance; PHI = Private Health Insurance.

A total of 10,254 individuals participated in the first wave of the survey conducted in 2006. Data on 8743 participants were included in the current study after removing data on 640 individuals registered with Medicare, 25 individuals with missing data regarding public health insurance, 22 individuals whose PHI registrations could not be confirmed, 816 individuals with missing data regarding annual household income, 6 individuals with missing data regarding education level, and 2 individuals with missing data regarding smoking habits (Figure 1).

### 2.2. Follow-Up Period and All-Cause Mortality

The follow-up period of the surviving panel was between the date of the first wave of the survey and the most recent participation date in the following editions. The follow-up period of the deceased panel was between the date of the first wave of the survey and the date of the individual’s death, which was confirmed via an interview with the deceased individual’s family. The all-cause mortality of participants was confirmed using the data from the deceased panels between the first and sixth waves.

### 2.3. Assessment of Covariates

The covariates were defined using the first wave of survey data from 2006. First, study participants were divided into two groups according to their insurance registration: those with NHI only, and those with both NHI and PHI. In terms of age, participants were divided into three groups (44–54 years; 55–64 years; and ≥65 years). Smoking status was classified as ‘current smoker’, ‘past smoker’, and ‘non-smoker’. Drinking status was either ‘yes’ for drinkers or ‘no’ for non-drinkers. For regular exercise status, participants were classified as ‘yes’ if they engaged in regular exercise (more than once a week) or ‘no’ if they did not. The past medical histories of participants included hypertension, diabetes mellitus, cancer, cardiovascular disease, and cerebrovascular disease. In terms of their education level, participants were divided into two groups: ≥high school education or <high school education. The economic status of participants was confirmed using annual household income and classified into four groups (<USD 10,000; USD 10,000–19,999; USD 20,000–29,999, and ≥USD 30,000). Marital status was classified as either ‘couple’ or ‘single’.

### 2.4. Statistical Analysis

The study participants were divided into two groups: those with NHI only and those with both NHI and PHI. A chi-squared test was conducted to assess the differences in sociodemographic characteristics between the two groups. The *t*-test was used to compare the follow-up observation periods of the two groups. Subsequently, results were adjusted for confounding variables using a Cox proportional hazard regression model, and mortality differences were shown as hazard ratios of the NHI–PHI group to the NHI group. The researchers additionally performed two subgroup analyses: one according to age (less than 55 years vs. 55 years or older) and one according to annual household income (using a threshold of USD 20,000). Cox proportional hazard models were used for the subgroup analyses. SPSS Statistics 18 (SPSS Inc., Chicago, IL, USA) was used to perform the statistical analyses.

## 3. Results

**Figure 2:**
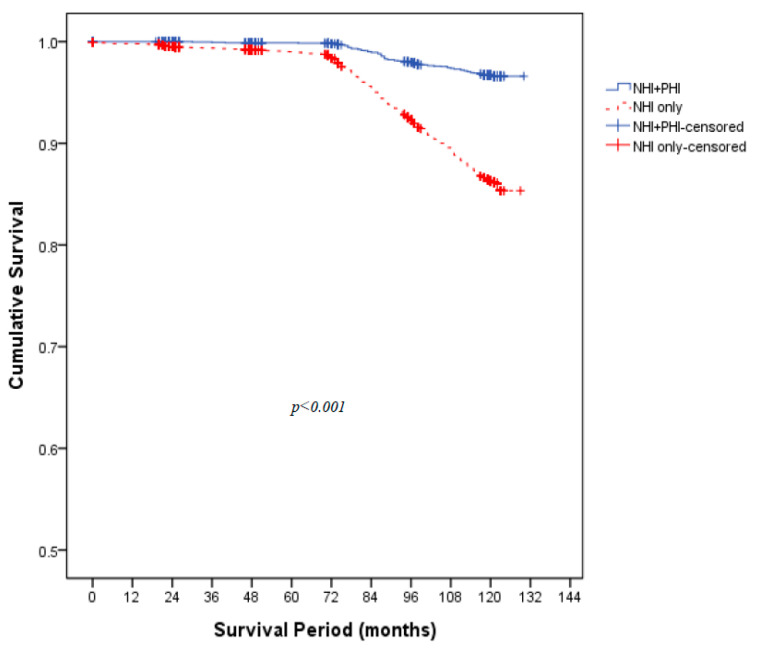
Kaplan–Meier survival plots compared by the log-rank test. Abbreviations: NHI = National Health Insurance; PHI = Private Health Insurance.

A total of 3004 individuals (34.4% of the study sample) had both NHI and PHI. The average follow-up period for the 8743 study participants was 94.5 months, and the average follow-up period of the NHI–PHI group was significantly longer than that of the NHI-only group (*p* < 0.001). The age and sex distributions differed between the two groups. In the NHI-only group, there were more men (*p* = 0.005) and participants tended to be older (*p* < 0.001). The NHI-only group also had a lower proportion of drinkers (*p* < 0.001) and people who regularly exercised (*p* = 0.003). For smoking status, there were no significant differences between the two groups (*p* = 0.118). For medical history, the NHI-only group had a higher proportion of participants with histories of hypertension (*p* < 0.001), diabetes mellitus (*p* < 0.001), cancer (*p* < 0.001), cardiovascular disease (*p* < 0.001), and cerebrovascular disease (*p* < 0.001). There were differences in levels of education and annual household income between the two groups, with the NHI–PHI group having a higher average education level (*p* < 0.001) and a higher average annual household income (*p* < 0.001) than the NHI-only group. The NHI-only group had a significantly higher proportion of non-married individuals (*p* < 0.001) (Table 1). The log-rank test confirmed that there were significant differences between the two groups for mortality (*p* < 0.001) (Figure 2).

The survival analysis using the Cox proportional hazard regression model also showed that the NHI–PHI group had a lower risk of mortality than the NHI-only group (Table 2). The subgroup analysis based on age showed that the NHI–PHI group had a lower risk of mortality than the NHI-only group in participants who were 55 years or older. Meanwhile, there were no significant differences in mortality between the NHI–PHI and NHI-only groups in participants who were less than 55 years old (Table 3). Moreover, additional PHI did not affect the mortality rate in participants with an annual household income of USD 20,000 or more, whereas the NHI–PHI group had a lower risk of mortality than the NHI-only group among participants with an annual household income of less than USD 20,000 (Table 4).

## 4. Discussion

This study found that mortality rates for people with PHI were lower than for those without PHI under Korea’s UHC system. Existing studies have also shown that health insurance status is related to mortality. Cheung et al. reported that all-cause mortality and all-cancer mortality increased for individuals without health insurance [12], and Lyon et al. also reported, in a study of critically ill patients, that mortality rates increased in the absence of health insurance [13]. Research has also found a protective association between mortality and health insurance in the case of infectious diseases [14,15].

Many studies have also reported that the type of insurance influences mortality. According to a study in the USA, mortality rates decreased in individuals with PHI compared to individuals with public health insurance or no health insurance [16,17]. Studies that analyzed mortality rates for specific causes of death showed similar results. A study of stroke patients conducted in the USA showed that those with PHI had higher rates of survival than those with public health insurance [18,19]. Similar findings related to cardiovascular disease have been shown. Two studies conducted in the USA reported that patients with PHI had lower mortality rates compared to those without insurance or with public health insurance [20,21]. With regard to cancer, Bittoni et al. showed that PHI lowered the all-cancer mortality rate in their study [16]. Studies on individual cancer types, including colorectal cancer [22], breast cancer [23], cervical cancer [24], lymphoma [25], hepatocellular carcinoma [26], and ovarian cancer [27], have also shown that patients with PHI had lower mortality rates. Results varied across studies in the case of death caused by injuries. Mehra et al. showed that the risk of death from injury decreases when one has PHI [28]. However, Jentzsch et al. reported that the insurance type did not have an impact on injury-related deaths [29].

Most of these studies confirmed that there were differences in the mortality rate between individuals with just public health insurance or no health insurance and individuals with PHI. As such, there are limitations to generalizing the findings of these studies across countries, especially with respect to Korea, where UHC has been achieved and almost the entire population has public health insurance. The nature of PHI under UHC is either complementary or supplementary. Complementary PHI coverage falls within the same scope as public health insurance coverage and covers costs due to the incompleteness of UHC. Supplementary PHI provides coverage for additional health services that are not covered under public health insurance. Korea operates a single public health insurance system, the NHI, and registration is compulsory. As of 2017, 97.2% of Koreans were registered with the NHI, and 2.8% were classified as Medical Aid beneficiaries and had all of their medical benefits covered by the state [30]. The NHI requires individuals to pay certain premiums, and there are tests and treatments not covered by the NHI. Therefore, PHI in Korea has both supplementary and complementary characteristics [31].

Considering these characteristics, this study was conducted to confirm whether additional PHI influenced the overall mortality rate of individuals registered with the NHI. Although there is little research on the relationship between PHI and mortality under a UHC system, the results of previous studies are in line with the findings of the present study. A USA study on the relationship between PHI and mortality among myocardial infarction patients showed that PHI decreased mortality despite the study subjects having coverage under Medicare, the USA public health insurance program for people aged 65 and older [32]. An Australian study examining end-stage kidney disease patients also showed that additional PHI decreased mortality [11]. PHI reduces the financial burden of additional tests, treatments, and rehabilitation outside the scope of public health insurance coverage; thus, patients are likely to become more active in seeking tests and treatments with additional PHI. Various studies have also shown that having PHI led to differences in how individuals used health services. In France, individuals with PHI used health services more frequently under their UHC system [33], and a Spanish study reported similar results [34].

To register for PHI, individuals need to be able to pay for insurance premiums. As a result, people with a higher SES are more likely to have additional PHI. In Korea, 37.4% of households have PHI in the bottom 20th percentile for household income, whereas the rate is 95.2% among households in the top 20th percentile [35]. A study in Taiwan, where a system similar to the Korean NHI is used, reported similar results [36]. Under UHC systems, the existence of health inequalities based on SES indicates that, although public health insurance guarantees that healthcare services are provided to anyone up to a certain level, this level is still not adequate. Therefore, the results of this study indicate that the NHI in Korea does not provide adequate medical services for its beneficiaries, and health inequalities occur due to SES. Furthermore, PHI registration is increasing in Korea, and the continuation of this trend is likely to result in wider health inequalities due to SES [37].

A strength of this study was that it took a longitudinal approach using data from a nationwide panel survey conducted over 10 years. Furthermore, this study confirmed the causal relationship between additional PHI and mortality by studying NHI-registered individuals in Korea, a country considered to have achieved UHC. Therefore, the results of this study can help health policy makers and experts to evaluate the effects of PHI in public health insurance systems where UHC has been achieved.

However, this study also has a number of limitations. This study was performed using secondary data, which were not designed and collected for this study. Therefore, a limitation of this study is that it did not use data that were specifically obtained for this study. For example, since the PHI data in KLoSA did not contain coverage-related information, it was not possible to analyze specific coverage-related data with regard to participants’ PHI. If an individual’s PHI coverage does not include a specific condition that the individual faces, PHI may not be helpful for the individual’s treatment or prognosis. In this case, however, the results examining the relationship between PHI and mortality would have been underestimated, rather than overestimated. In addition, since the KLoSA did not collect data on variables related to health disparities such as health beliefs, those variables were not included in the analysis [21]. As such, these limitations should be considered when interpreting the findings of this study. Another limitation is the possibility that differences in health status may exist between individuals with and without PHI. It is possible that those without PHI may not be able to secure PHI due to their medical histories, as opposed to financial strain or individual choice. In Korea, the majority of insurance companies request health checkup results and past medical records prior to PHI registration. Consequently, poor checkup results and a history of diseases can lead to rejected PHI applications. As such, healthy individuals are more likely to have additional PHI, resulting in a lower mortality rate among people with additional PHI. This study also found that the prevalence rates of hypertension, diabetes, cancer, cardiovascular disease, and cerebrovascular disease were lower among those with PHI. Medical history was, however, added as a covariate in the analysis, and it is thought that the influence of the disparity between prevalence rates would have been significantly adjusted for at this stage of the analysis.

## 5. Conclusions

The results of the present study indicate that individuals covered by Korean NHI who have additional PHI have a lower mortality rate than individuals who are only covered by Korean NHI and have no additional PHI. In particular, the finding that having additional PHI did not affect the mortality rate in high-income individuals indicates that health disparities associated with having additional PHI were notable in the low-income group. Therefore, policy makers and health experts should improve the system by reducing individual premiums and adding coverage for new health technologies and new drugs in order to resolve health disparities related to SES and PHI.

## Figures and Tables

**Table 1:** Characteristics of the study sample according to private health insurance status.

Variables	Total	NHI + PHI	NHI Only	*p*-Value
	(*n* = 8743)	(*n* = 3004)	(*n* = 5739)	
Follow-up period (months)	94.5 ± 43.0	99.3 ± 41.1	92.0 ± 43.7	<0.001
Sex				0.005
Male	3892 (44.5)	1275 (42.4)	2617 (45.6)	
Female	4851 (55.5)	1729 (57.6)	3122 (54.4)	
Age (years)				<0.001
≥65	3276 (37.5)	273 (9.1)	3003 (52.3)	
55–64	2497 (28.6)	911 (30.3)	1586 (27.6)	
45–54	2970 (34.0)	1820 (60.6)	1150 (20.0)	
Smoking status				0.118
Current smoker	1692 (19.4)	574 (19.1)	1118 (19.5)	
Past smoker	839 (9.6)	263 (8.8)	576 (10.0)	
Non-smoker	6212 (71.1)	2167 (72.1)	4045 (70.5)	
Drinking status				0.003
Yes	3354 (38.4)	1407 (46.8)	1947 (33.9)	
No	5389 (61.6)	1597 (53.2)	3792 (66.1)	
Regular exercise status				<0.001
Yes	2353 (26.9)	866 (28.8)	1487 (25.9)	
No	6390 (73.1)	2138 (71.2)	4252 (74.1)	
Hypertension				<0.001
Yes	2284 (26.1)	541 (18.0)	1743 (30.4)	
No	6459 (73.9)	2463 (82.0)	3996 (69.6)	
Diabetes mellitus				<0.001
Yes	978 (11.2)	235 (7.8)	743 (12.9)	
No	7765 (88.8)	2769 (92.2)	4996 (87.1)	
Cancer				<0.001
Yes	199 (2.3)	41 (1.4)	158 (2.8)	
No	8544 (97.7)	2963 (98.6)	5581 (97.2)	
Cardiovascular disease				<0.001
Yes	386 (4.4)	65 (2.2)	321 (5.6)	
No	8357 (95.6)	2939 (97.8)	5418 (94.4)	
Cerebrovascular disease				<0.001
Yes	279 (3.2)	40 (1.3)	239 (4.2)	
No	8464 (96.8)	2964 (98.7)	5500 (95.8)	
Education level				<0.001
≥High school	3413 (39.0)	1679 (55.9)	1734 (30.2)	
<High school	5330 (61.0)	1325 (44.1)	4005 (69.8)	
Annular household income (USD)				<0.001
≥30,000	2332 (26.7)	1175 (39.1)	1157 (20.2)	
20,000–29,999	1335 (15.3)	537 (17.9)	798 (13.9)	
10,000–19,999	1687 (19.3)	538 (17.9)	1149 (20.0)	
<10,000	3389 (38.8)	754 (25.1)	2635 (45.9)	
Marital status				<0.001
Single	1648 (18.8)	286 (9.5)	1362 (23.7)	
Couple	7095 (81.2)	2718 (90.5)	4377 (76.3)	
Overall death				<0.001
Living	8075 (92.4)	2924 (97.3)	5151 (89.8)	
Deceased	668 (7.6)	80 (2.7)	588 (10.2)	

NHI = National Health Insurance; PHI = private health insurance. Numbers in parentheses indicate percentages. *p*-values were calculated using the chi-squared test for categorical variables and the *t*-test for continuous variables (follow-up period).

**Table 2:** Hazard ratios of death according to private health insurance status ^a^.

	NHI Only	NHI + PHI
HR	1	0.53 *
95% CI	Reference	(0.41–0.69)

HR = hazard ratio; CI = confidence interval; NHI = National Health Insurance; PHI = private health insurance. ^a^ HRs were calculated by the Cox proportional hazard regression model. * Adjusted for sex, age, smoking status, drinking status, regular exercise status, hypertension, diabetes, cancer, cardiovascular disease, cerebrovascular disease, education level, annual household income, and marital status.

**Table 3:** Hazard ratios of death according to private health insurance status by age ^a^.

	<55 Years		≥55 Years	
	HR	95%	HR	95% CI
NHI only	1	Reference	1	Reference
NHI + PHI	0.89 *	(0.25–1.13)	0.33 *	(0.24–0.45)

HR = hazard ratio; CI = confidence interval; NHI = National Health Insurance; PHI = private health insurance. ^a^ HRs were calculated by the Cox proportional hazard regression model. * Adjusted for sex, smoking status, drinking status, regular exercise status, hypertension, diabetes, cancer, cardiovascular disease, cerebrovascular disease, education level, annual household income, and marital status.

**Table 4:** Hazard ratios of death according to private health insurance status by annual household income ^a^.

	<USD 20,000		≥USD 20,000	
	HR	95%	HR	95% CI
NHI only	1	Reference	1	Reference
NHI + PHI	0.52 *	(0.41–0.67)	0.81 *	(0.56–1.20)

HR = hazard ratio; CI = confidence interval; NHI = National Health Insurance; PHI = private health insurance. ^a^ HRs were calculated by the Cox proportional hazard regression model. * Adjusted for sex, age, smoking status, drinking status, regular exercise status, hypertension, diabetes, cancer, cardiovascular disease, cerebrovascular disease, education level, and marital status.

## Data Availability

The data used to support the findings of this study were supplied under license and so cannot be made freely available. Requests for access to these data should be made to jiomoris@naver.com.

## References

[1] Sasaki T., Izawa M., Okada Y. (2015). Current trends in health insurance systems: OECD countries vs. Japan. Neurol. Med. Chir..

[2] Dronina Y., Yoon Y.M., Sakamaki H., Nam E.W. (2016). Health system development and performance in Korea and Japan: A comparative study of 2000–2013. J. Lifestyle Med..

[3] Sustainable Health Financing, Universal Coverage and Social Health Insurance. https://apps.who.int/iris/handle/10665/20383.

[4] Baggio S., Dupuis M., Wolff H., Bodenmann P. (2018). Associations of lack of voluntary private insurance and out-of-pocket expenditures with health inequalities. Evidence from an international longitudinal survey in countries with universal health coverage. PLoS ONE.

[5] Kim S.W. (2016). Policy Review of Health Insurance Coverage Expansion.

[6] Jin Y., Hou Z., Zhang D. (2016). Determinants of health insurance coverage among people aged 45 and over in China: Who buys public, private and multiple insurance. PLoS ONE.

[7] LaPar D.J., Bhamidipati C.M., Mery C.M., Stukenborg G.J., Jones D.R., Schirmer B.D., Kron I.L., Ailawadi G. (2010). Primary payer status affects mortality for major surgical operations. Ann. Surg..

[8] Gabriel L.E., Bailey M.J., Bellomo R., Stow P., Orford N., McGain F., Santamaria J., Scheinkestel C., Pilcher D.V. (2016). Insurance status and mortality in critically ill patients. Crit. Care Resusc..

[9] Sobotka L.A., Hinton A., Conteh L.F. (2019). Insurance status impacts treatment for hepatocellular carcinoma. Ann. Hepatol..

[10] Singh J.A., Cleveland J.D. (2020). Insurance payer type and patient income are associated with outcomes after total shoulder arthroplasty. J. Rheumatol..

[11] Sriravindrarajah A., Kotwal S.S., Sen S., McDonald S., Jardine M., Cass A., Gallagher M. (2020). Impact of supplemental private health insurance on dialysis and outcomes. Intern. Med. J..

[12] Cheung M.R. (2013). Lack of health insurance increases all cause and all cancer mortality in adults: An analysis of national health and nutrition examination survey (NHANES III) data. Asian Pac. J. Cancer Prev..

[13] Lyon S.M., Benson N.M., Cooke C.R., Iwashyna T.J., Ratcliffe S.J., Kahn J.M. (2011). The effect of insurance status on mortality and procedural use in critically ill patients. Am. J. Respir. Crit. Care Med..

[14] Younossi Z.M., Otgonsuren M., Henry L., Arsalla Z., Stepnaova M., Mishra A., Venkatesan C., Hunt S. (2015). Inpatient resource utilization, disease severity, mortality and insurance coverage for patients hospitalized for hepatitis C virus in the United States. J. Viral Hepat..

[15] Jabs A.W., Jabs D.A., Van Natta M.L., Palella F.J., Meinert C.L. (2018). Insurance status and mortality among patients with aids. HIV Med..

[16] Bittoni M.A., Wexler R., Spees C.K., Clinton S.K., Taylor C.A. (2015). Lack of private health insurance is associated with higher mortality from cancer and other chronic diseases, poor diet quality, and inflammatory biomarkers in the United States. Prev. Med..

[17] Saunders M.R., Ricardo A.C., Chen J., Chin M.H., Lash J.P. (2016). Association between insurance status and mortality in individuals with albuminuria: An observational cohort study. BMC Nephrol..

[18] Hoffmeister L., Lavados P.M., Murta-Nascimento C., Araujo M., Olavarría V.V., Castells X. (2013). Short- and long-term survival after stroke in hospitalized patients in Chile: A nationwide 5-year study. J. Stroke Cerebrovasc. Dis..

[19] McManus M., Ovbiagele B., Markovic D., Towfighi A. (2015). Association of insurance status with stroke-related mortality and long-term survival after stroke. J. Stroke Cerebrovasc. Dis..

[20] LaPar D.J., Stukenborg G.J., Guyer R.A., Stone M.L., Bhamidipati C.M., Lau C.L., Kron I.L., Ailawadi G. (2012). Primary payer status is associated with mortality and resource utilization for coronary artery bypass grafting. Circulation.

[21] Ng D.K., Brotman D.J., Lau B., Young J.H. (2012). Insurance status, not race, is associated with mortality after an acute cardiovascular event in Maryland. J. Gen. Intern. Med..

[22] Ellis L., Canchola A.J., Spiegel D., Ladabaum U., Haile R., Gomez S.L. (2018). Trends in cancer survival by health insurance status in California from 1997 to 2014. JAMA Oncol..

[23] Hsu C.D., Wang X., Habif D.V., Ma C.X., Johnson K.J. (2017). Breast cancer stage variation and survival in association with insurance status and sociodemographic factors in US women 18 to 64 years old. Cancer.

[24] Jalloul R.J., Sharma S., Tung C.S., O’Donnell B., Ludwig M. (2018). Pattern of care, health care disparities, and their impact on survival outcomes in stage IVB cervical cancer: A nationwide retrospective cohort study. Int. J. Gynecol. Cancer.

[25] Han X., Jemal A., Flowers C.R., Sineshaw H., Nastoupil L.J., Ward E. (2014). Insurance status is related to diffuse large B-cell lymphoma survival. Cancer.

[26] Hoehn R.S., Hanseman D.J., Jernigan P.L., Wima K., Ertel A.E., Abbott D.E., Shah S.A. (2015). Disparities in care for patients with curable hepatocellular carcinoma. HPB.

[27] Balli S., Fey M.F., Hänggi W., Zwahlen D., Berclaz G., Dreher E., Aebi S. (2000). Ovarian cancer. An institutional review of patterns of care, health insurance and prognosis. Eur. J. Cancer.

[28] Mehra T., Moos R.M., Seifert B., Bopp M., Senn O., Simmen H.P., Neuhaus V., Ciritsis B. (2017). Impact of structural and economic factors on hospitalization costs, inpatient mortality, and treatment type of traumatic hip fractures in Switzerland. Arch. Osteoporos..

[29] Jentzsch T., Neuhaus V., Seifert B., Osterhoff G., Simmen H.P., Werner C.M., Moos R. (2016). The impact of public versus private insurance on trauma patients. J. Surg. Res..

[30] (2017). National Health Insurance Statistics. https://www.hira.or.kr/bbsDummy.do?pgmid=HIRAA020045020000&brdScnBltNo=4&brdBltNo=2310.

[31] OECD Health Statistics (2018). Definitions, Sources and Methods. https://www.oecd.org/els/health-systems/Table-of-Content-Metadata-OECD-Health-Statistics-2018.pdf.

[32] Fang J., Alderman M.H. (2004). Does supplemental private insurance affect care of medicare recipients hospitalized for myocardial infarction?. Am. J. Public Health.

[33] Buchmueller T.C., Couffinhal A., Grignon M., Perronnin M. (2004). Access to physician services: Does supplemental insurance matter? Evidence from France. Health Econ..

[34] Rocha K.B., Rodríguez-Sanz M., Pérez K., Obiols J.E., Borrell C. (2013). Inequalities in the utilization of psychiatric and psychological services in Catalonia: A multilevel approach. Adm. Policy Ment. Health.

[35] Kim J.M. (2017). The Analysis of Disparity in Private Health Insurance and Normalization Methods.

[36] Liu T.C., Chen C.S. (2002). An analysis of private health insurance purchasing decisions with national health insurance in Taiwan. Soc. Sci. Med..

[37] Shin H.W., Lim J.W. (2018). Current state of private insurance management in OECD member states—Focusing on the relationship between public and private insurances. HIRA.

